# Interactions of hydrology, geochemistry, and biodiversity in woodland ponds located in riverine floodplains: case study from Scotland

**DOI:** 10.1007/s11356-023-27890-6

**Published:** 2023-06-22

**Authors:** Vladimir Krivtsov, Jim Buckman, Steve Birkinshaw, Valerie Olive

**Affiliations:** 1 Edinburgh University, Edinburgh, Scotland UK; 2 Royal Botanic Garden Edinburgh, Edinburgh, Scotland UK; 3 Heriot Watt University, Edinburgh, Scotland UK; 4 Newcastle University, Newcastle, England UK; 5 SUERC, University of Glasgow, Glasgow, Scotland UK

**Keywords:** Ecological monitoring, Environmental modelling, Vegetation, Biological community, Climate change resilience, Newts habitat suitability, Green infrastructure, Nature-based solutions, Geochemical patterns

## Abstract

The Gore Glen pond is located in the forested floodplain of the Gore River, in the Mid-Lothian region near Edinburgh, Scotland. This work has considered in detail the hydrology, hydrochemistry, and biodiversity of the pond together with all their interactions and in addition interactions with the adjacent Gore River. It is important as it is one of few studies considering all these issues for a pond located in a riverine floodplain. This work shows the pond is connected to the Gore River for discharge events in the river larger than a 1 in 5-year return period. For these events, the pond acts as an online storage feature and will attenuate the flow, thus contributing towards the mitigation of downstream flooding. These large events are also a potential source of the large amount of sediments, as well as adsorbed chemicals, accumulated in the pond. The open water surface of the pond is covered by duckweed, *Lemna minor*, for most of the year, and that has profound implications for the hydrobiological community and biogeochemical cycling. The system is characterised by nutrient release from sediments due to the extensive hypolimnion anoxia in the summer/autumn period caused by the decomposition of organic matter. Algae are mostly represented by epiphytic diatoms and the cyanobacterium *Microcystis*, but their development appears to be constrained by light penetration. The eutrophic conditions are also conducive for the population of *Difflugia* (Protozoa, Rhizopoda). This work improves our understanding of the ecological relations of testate amoebae—an important group which has recently been used as biological indicators for the analysis of paleoecological relationships and paleogeographical reconstructions.

**Supplementary Information:**

The online version contains supplementary material available at 10.1007/s11356-023-27890-6.

## Introduction

Ponds in riverine floodplains contribute to local biodiversity and provide a number of further important ecosystem functions related to recreation and alleviation of flood risk amongst others (CIRIA 2019; Hill et al. 2016; Tockner et al. 2010). Multiple benefits provided by these sites are best understood through a detailed insight into interlinked ecological patterns, in particular, those related to hydrology, hydrochemistry, and biodiversity.

Although the value of ponds for alleviation of flood risk and improvements in water quality have long been recognised, multiple benefits provided by ponds have only recently started to be appreciated, and case studies offering a detailed interdisciplinary investigation are rather rare (Krivtsov et al. 2022). Studies tend to focus on a limited number of ecological issues relevant to the provision of ecosystem services or cover a wider ground without going into specific details (Alves et al. 2020; Jose et al. 2015; Oertli and Parris 2019). Consequently, the scientific evidence for environmental management of these important assets remains limited (Hassall et al. 2016). In particular, for the online storage ponds located in riverine floodplains (Quinn et al. 2022), there appears to be a scarcity of the detailed insight into ecological patterns and their interactions with flow regime and geochemical settings.

Testate amoebae, and in particular *Difflugia*, as well as other agglutinated testate amoebae, have been previously investigated as potential ecological indicators for environmental conditions in lakes using analysis of geological cores, but it has been acknowledged that further practical applications are hampered by the scarcity of knowledge on their contemporary ecological associations (Prentice et al. 2018). Furthermore, recent research provided some (albeit limited) information on the occurrence and ecological interactions in urban ponds (Krivtsov et al. 2020b, c), but investigations in woodland floodplain ponds are lagging behind.

This work focuses on a woodland pond located in the floodplain of Gore Water river (Scotland, UK) and aims to further the understanding of its overall functioning and provide guidance on the best management actions of this and similar sites in other riverine floodplains, an area in which there are gaps in the current research. This interdisciplinary aim is achieved by carrying out a range of physical, chemical, and biological measurements combined with hydrological and hydrodynamic modelling of the pond and its interaction with the Gore Water. The objective of this is to understand the hydrology, hydrochemistry, and biodiversity aspects of the pond in more detail and so help in providing insights on the interconnections between these aspects. In addition, we consider in detail the testate amoebae in the pond in view of their potential use as ecological indicators.

## Materials and methods

### Site description

**Fig. 1:**
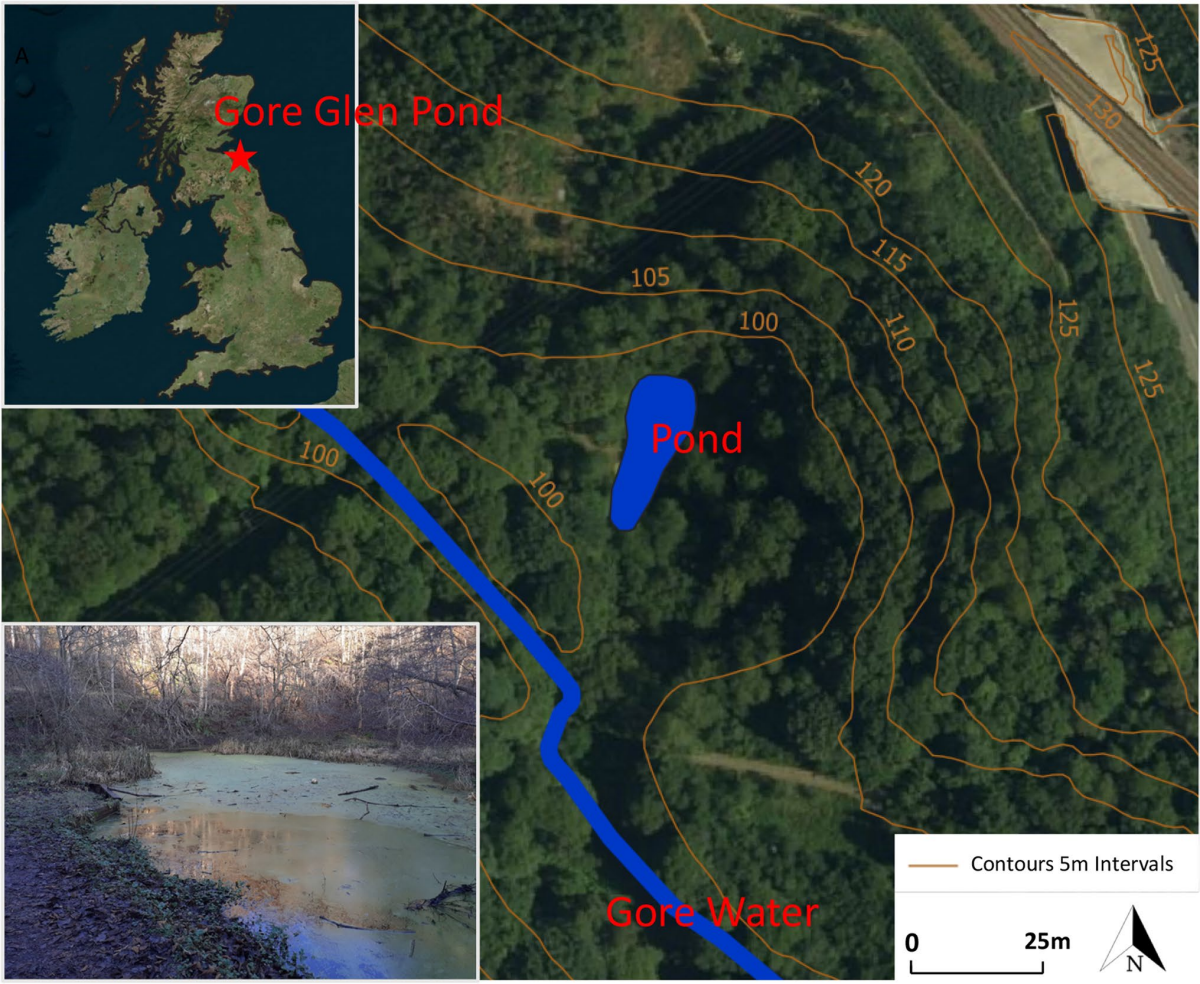
Showing the Gore Glen pond and the Gore Water (which runs from the south-east to the north-west). The insets show the location of the pond and an image of the pond from December 2019

The woodland pond is located in the floodplain of Gore Water situated within a local biodiversity site (LBS) west of Gorebridge (Midlothian, Scotland, UK). The pond is within the area of Gore Glen Country Park managed by the Midlothian Ranger Service (Fig. 1). There are additional details of the pond’s location in previous research carried out at the site (Jarvie et al. 2017; Krivtsov et al. 2019; Krivtsov et al. 2020a).

The surface area of 500 m^2^ was reported by (Jarvie et al. 2017), whilst our research shows that the surface area and volume are actually variable. The same authors also stated that creation of the pond dates back to 1794–1861 and was related to coal mining operations in the area. Older maps show connections of the pond to the river; but on inspection, these are not current, and the pond appears to have not received any surface inflow for the last few years.

### Hydrological assessment and modelling

There are no discharge measurements available for the Gore Water. However, the Gore Water catchment is nested within the catchment of South Esk at Prestonholm, so using standard methods (Kjeldsen et al. 2008), it is possible to estimate the peak discharges for the Gore Water from the annual flow maxima of the South Esk (available for the period 1963–1989). For the closely related catchments, the annual max discharge is proportional to the area^0.85^; hence, given the area of the Gore Water catchment (33.2 km^2^) and the South Esk catchment at Prestonholm (112 km^2^), annual maxima discharges for Gore Water catchment can be worked out for a range of return periods detailed in Table 1.

**Table 1:** Estimated maximum discharges at Gore Water (m^3^/s) used as inflow boundary conditions for the CityCAT simulations. Maximum discharge estimates are given for 1 in 2-year (Q2), 5-year (Q5), 10-year (Q10), 20-year (Q20), 50-year (Q50), and 100-year (Q100) return periods

Return period	Discharge (m^3^/s)
Q2	6.80
Q5	11.53
Q10	15.73
Q20	20.39
Q50	24.80
Q100	29.72

Hydrological and hydrodynamic modelling analyses were carried out, respectively, using the SHETRAN (Ewen et al. 2000) and CityCAT models (Glenis et al. 2018). The SHETRAN model is a continuous simulation hydrological model for surface and subsurface flows, and the modelling was carried out for a domain including the pond and the hillslope that drains into the pond. It produces a continuous time series of the surface water level or water table depth in each grid square and so enables a time series of the pond’s surface area to be produced. It was setup to not consider the hydraulic connection between the pond and the river. The CityCAT hydrodynamic model is an event based model that simulates two-dimensional surface water flows. The domain used (Fig. 1) includes a section of the Gore Water plus the hillslope that drains into the pond. The model is used to simulate the hydraulic connections between the Gore River and the pond under extreme events (Fenner et al. 2019; O'Donnell et al. 2020).

Both models require detailed DEM data which were obtained from the 2 m resolution Scottish Public Sector LiDAR (Phase I) dataset. These data were used directly in the CityCAT model domain (which gives a 285 by 282 grid). SHETRAN uses a 5-m grid resolution based on the 2 m LIDAR data. This gives 122 by 118 vertical columns with 20 cells in each column containing the soil and geology information (the model has a limit of 200 by 200 vertical columns).

SHETRAN simulations were run for 19 months from 1 January 2018 to 31 July 2019 using daily Scottish Environment Protection Agency rainfall for the area. Data on the potential evaporation used in the simulations was obtained from the CHESS dataset (Robinson et al. 2016; Robinson et al. 2017). Appropriate parameter values for the area’s geology, soils and land use were obtained from standard libraries (Lewis et al. 2018).

CityCAT simulations were carried out for the 6 extreme events specified in Table 1, with the discharges used as inflow boundary conditions applied where the river enters the domain on its southern boundary. CityCAT uses the Green-Ampt equations (Green and Ampt 1911) to calculate infiltration based on the soil porosity, hydraulic conductivity, and suction head (Chow et al. 1988; Kutílek and Nielsen 1994; Warrick 2003). Whether water is able to infiltrate at any particular grid cell depends on whether the soil moisture content is already at field capacity. When the soil is saturated with moisture, any additional precipitation contributes to surface runoff. The simulations presented in this paper relate to the dry initial conditions.

### Data collection and analysis

Information on the biological community of the site has been obtained through a series of regular ecological surveys variously carried out between 2018 and 2022. Both vascular plants and bryophytes were recorded in and around the pond in a variety of habitats, including deciduous woodland, woodland edge, open water, marginal habitat, and unpaved path. A mycological survey of both lichenised fungi and non-lichenised fungi was carried out in the area around the pond. Observations of vertebrates at the site were restricted to birds (recorded by sight or song) and amphibians (visual observations and netting). The biodiversity records are currently being deposited with The Wildlife Information Centre (TWIC) in publicly available databases.

Abundances of planktonic organisms and macroinvertebrates were estimated by standard methods. Aquatic macroinvertebrates were sampled by 3 minutes sweep sampling, whilst plankton samples were obtained using a phytoplankton net (Krivtsov et al. 2020b). For the assessment of macroinvertebrate diversity, Walley Hawkes Paisley Trigg (WHPT) scores and the average score per taxon (ASPT) were estimated using a Microsoft Excel spreadsheet supplied by the Freshwater Biological Association (FBA).

The samples were analysed using light and scanning electron microscopy as detailed in (Buckman and Krivtsov 2022). Measurements of water quality parameters were carried out using standard field equipment. The levels of pH, general hardness (GH), carbonate hardness (KH), electrical conductivity (EC), nitrates, nitrites, and chlorine (Cl) were measured in the field using indicator strips JBL EasyTest 6in1. The levels of oxygen were measured using a Lutron PDO-519 probe. Measurements of pH in the collected water samples were also carried out in the lab using standard lab equipment. Data collection was carried out as part of a wider monitoring programme of ponds in Edinburgh and the Lothians (Krivtsov et al. 2019). Hence, the selection of variables was, in part, influenced by practical and logistical considerations, including the speed of analysis and the availability of equipment.

Concentrations of chemical elements in the samples of pond water were assessed using an inductively coupled plasma mass spectrometry (ICP MS) at the University of Glasgow. The method allows simultaneous analysis of >50 chemical elements. Here, we present summary results for Ag, Al, Ba, Co, Cr, Eu, Fe, Mn, Mg, P, Si, and Li. The selection of elements was determined by the equipment capabilities and by the usefulness of the results obtained for the interpretation of the ecological patterns observed. Data processing and statistical analysis were carried out using Microsoft Excel and Matlab. Those elements whose levels were frequently below the detection threshold and were excluded from statistical analysis. Further information on the methods used is available in our previous publications (Krivtsov et al. 2019, 2020a, b, c).

## Results

### Hydrology

Under normal conditions, the pond has no active connection with the river. Under these conditions, the hydrological dynamics of the pond has been simulated using the SHETRAN model. This demonstrates that the surface water level of the pond reflects that of the ground water (data not shown). Both the volume and the surface area of the pond increase following periods of intense precipitation, fed by ground water ingress and direct precipitation. The resulting inundation of the adjacent area provides a valuable buffering mechanism for alleviation of flood risk, as well as an important mesic habitat hosting a number of biological taxa.

**Fig. 2:**
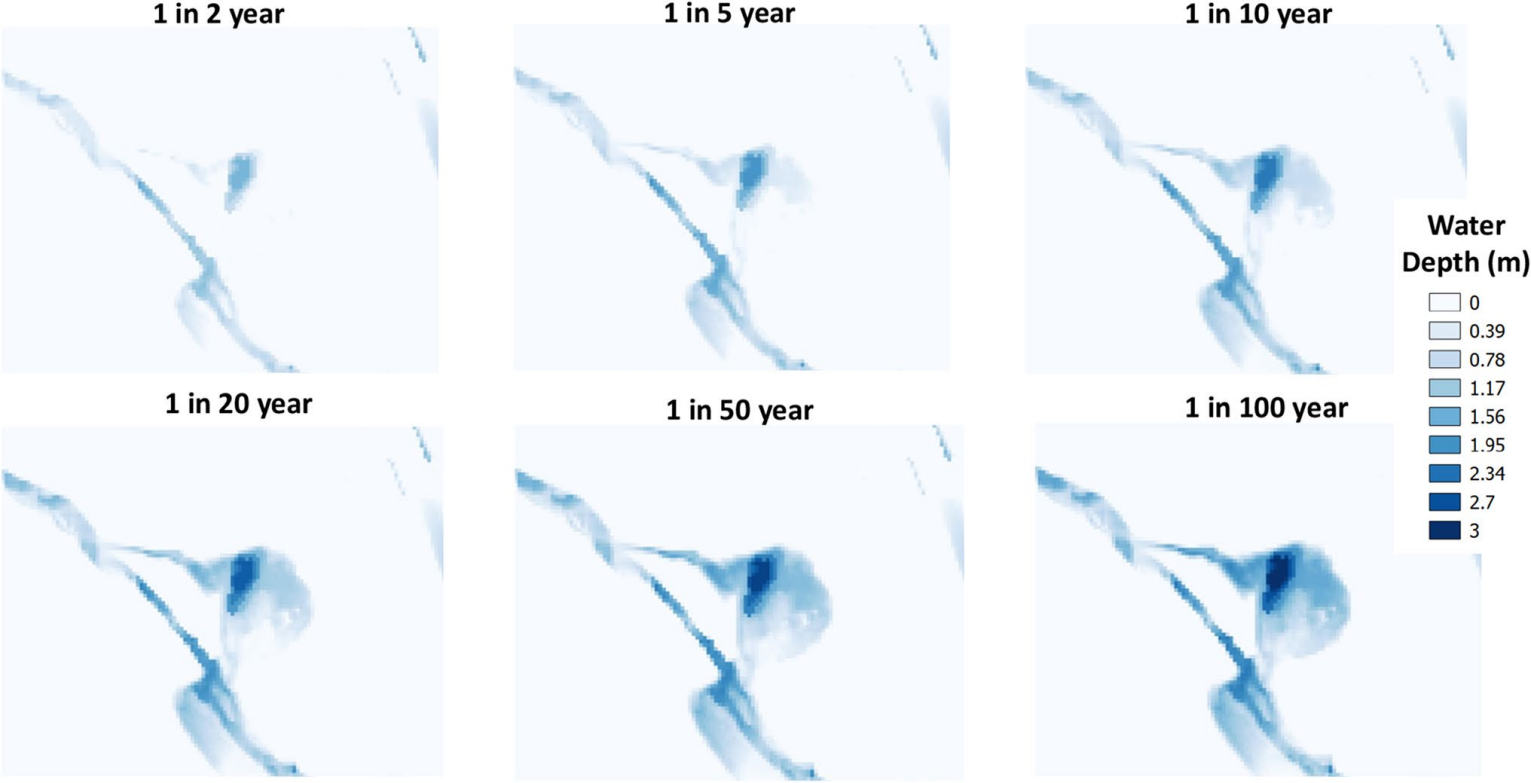
Water depths from the CityCAT simulations with inflow boundary conditions applied to the southern boundary where the Gore Water enters the domain. Note that for these extreme events, there is a connection between the pond and the river for events more extreme than 1 in 5 year

Under extreme events, the CityCAT hydrodynamic model is used to analyse the reconnection between the pond and the river via the surface inflow and outflow (the results of a simulation where this occurs are presented in a supplementary video). The results show that the pond reconnects with the river for events more extreme than 1 in 5 years (Fig. 2), with a river discharge greater than 11.5 m^3^/s (Table 1). It is also evident that the re-establishment of the inflow happens before the re-establishment of the outflow, and that both the area and the water depth increase with the extremity of the event. For these extreme high flow events the pond, therefore, effectively serves as an online storage pond and a flood attenuation feature (Quinn et al. 2022), even though it has not been specifically designed as such.

Although the modelling work suggests the pond reconnects with the river for flood events more extreme than 1 in 5 years, there is uncertainty related to this value. The local rangers, who visit the site regularly, have seen no indication of it occurring and suggest it only happens for even more extreme events. There are likely to be errors in the measured DEM values due to the mature trees that cover the majority of the domain, and this may explain the discrepancy between the modelling results and the observed reconnection of the pond to the river. However, the modelling work uses the best available data so it is been kept, whilst acknowledging the uncertainty in the result.

### Hydrochemistry

**Fig. 3:**
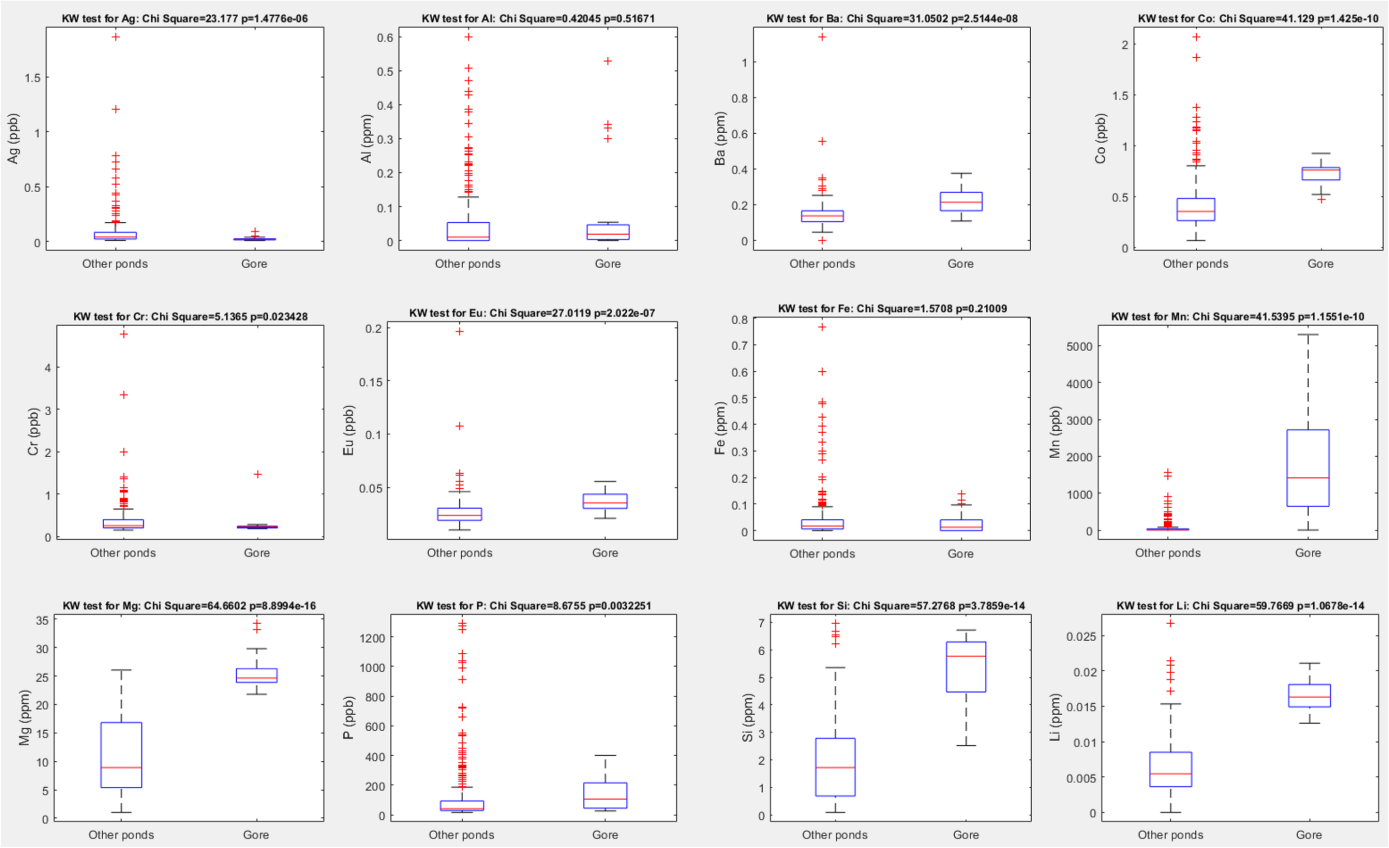
Comparison of the levels for selected chemical elements in Gore Glen and other ponds

**Fig. 4:**
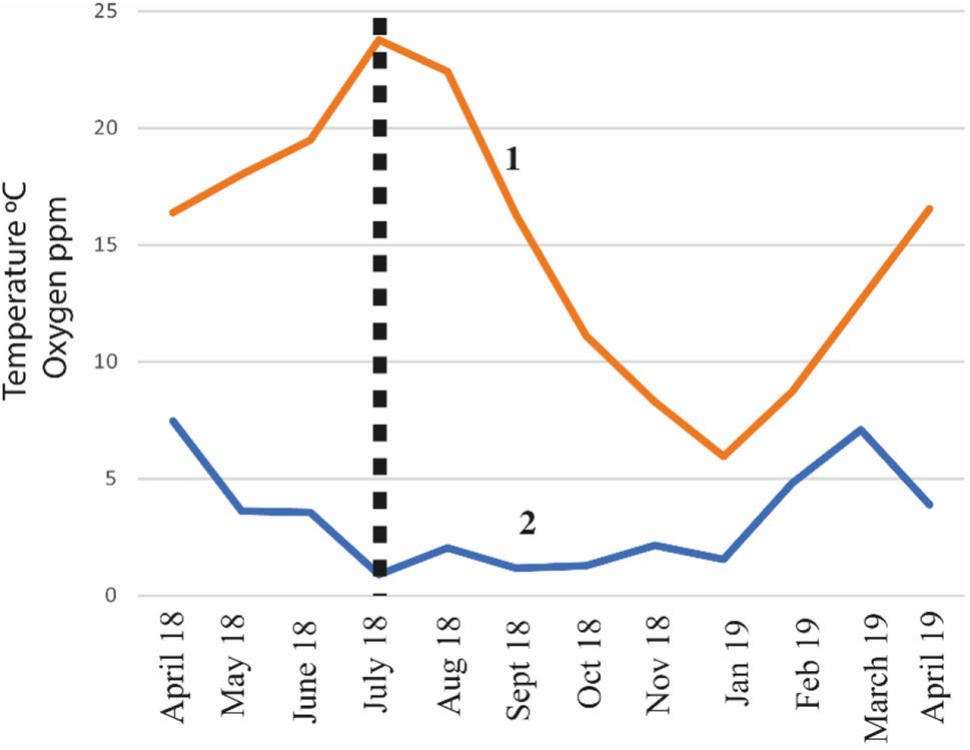
Graph of temperature and oxygen change at Gore Glen pond. 1 = temperature profile, 2 = oxygen profile. Note that the peak temperature occurs in July (dashed line), which equates with a period of reduced oxygenation, and high *Difflugia* productivity (see Testate amoeba case study section)

As the pond site occupies a disused colliery, its industrial past is reflected in the observed levels of certain chemicals (Fig. 3). The bottom has a very thick layer of unconsolidated sediments, which have accumulated a large storage of pollutants (Krivtsov et al. 2020a). The levels of GH and KH are higher, whilst nitrate concentrations are lower than in urban ponds (all measured using indicator strips JBL EasyTest 6in1). Oxygen becomes depleted throughout the summer/early autumn period (Fig. 4). Water concentrations (ICP measurements) of certain elements (e.g. P, Al, Fe, Mn) increase in summer due to anoxia, and pyrite framboids have been observed using SEM (Krivtsov et al. 2019). However, unlike the urban ponds studied in parallel research, Gore Glen does not appear to be affected by road runoff, which is reflected in smaller Cl levels.

### Biodiversity

The biodiversity benefits from the inter-zonal effect (ecoton). The pond is surrounded by a mature woodland containing 86 species of vascular plants in the area close to the pond (Appendix Table 2). Overall, there are >100 plant and >40 fungal species occurring in the immediately adjacent area. In summer, practically all of the open water area is covered by duckweed *Lemna minor* L., which restricts light penetration. Despite that, the aquatic habitats feature a number of phytoplankton species, including 15 diatoms, 5 cyanobacteria, 17 chlorophytes (including euglenophytes), and 1 dinoflagellate; 14 zooplankton taxa have also been observed.

**Fig. 5:**
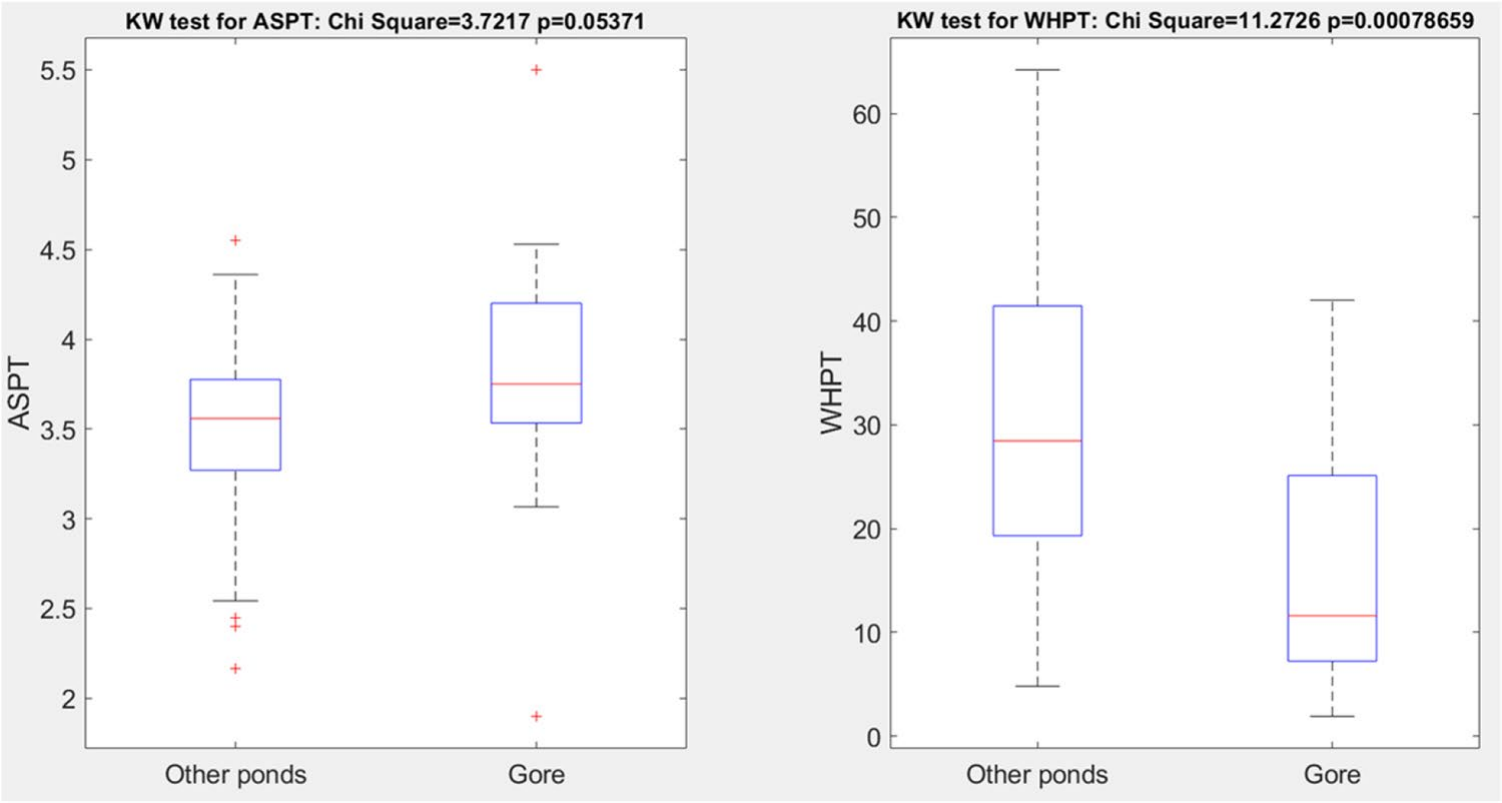
Comparison for macroinvertebrate indices of biological water quality in Gore Glen and other (predominantly urban) ponds in Edinburgh and Lothian region of Scotland. “KW,” Kruskal-Wallis test

The macroinvertebrate biodiversity indices used in this study (Fig. 5, Appendices Tables 3 and 4) are commonly applied for estimating the influence of pollution on the aquatic environment (Chadd 2010). The macroinvertebrate diversity in Gore Glen appears to be impoverished (WHPT index = 16.5) and features the presence of animals capable to withstand eutrophic conditions with low oxygen levels (e.g., *Radix baltica* and *Chironomidae*). Coleoptera is mainly represented by very small genera of *Haliplidae* and *Dytiscidae* families*.* Fringe habitats and the macrophytes growing in the near-shore zone are important for the macroinvertebrates present.

It should be noted, however, that the ASPT index (commonly regarded as the main indicator of water quality) is actually sub-significantly higher than in urban ponds studied in areas around Edinburgh (i.e., significant on the 94% probability level, *p* < 0.06)—see Fig. 5. Furthermore, the pond supports a breeding population of palmate newts *Lissotriton helveticus*. This exemplifies the importance of the availability of diverse habitats within the woodland setting for those species who need access to both terrestrial and aquatic environment to complete their life cycle.

The phytoplankton of the pond is dominated by cyanobacteria, mainly *Microcystis* and (to a lesser degree) *Oscillatoria*. Diatoms, mainly *Epithemia*, are also commonly present and occur mainly as epiphytes on duckweed *Lemna minor*. It should be noted, however, that the phytoplankton growth appears to be restricted by the limited light penetration (because of lush *Lemna minor* growth and also elevated concentrations of suspended particulates). However, when in summer green algae contribute significantly to the mixed phytoplankton bloom, chlorophyll levels are comparable (and may even exceed) those registered in a eutrophic phytoplankton-dominated lake (Krivtsov et al. 2001a; Tien et al. 2002).

The zooplankton community is characterised by the prominence of testate amoebae, in particular, *Difflugia*, which was frequent in the samples taken in the summer and early autumn (Krivtsov et al. 2021). The population explosion appears to happen between May (when only 1 specimen was observed on filter papers analysed by SEM) and July (when 75 specimens were observed). This is in contrast to a number of other ponds studied around Edinburgh and the Lothians, where *Difflugia* appear to be far less prominent. Other protozoans, in particular ciliates and small heterotrophic flagellates, were also regularly observed. Amongst larger zooplankton, rotifers were rather scarce, whilst such crustaceans as *Cyclops* and *Chydorus* were rather frequent in the samples; however, there was a remarkable scarcity of *Daphnia*. Other noteworthy (in terms of their relative abundance) microbiota recorded at Gore Glen include chrysophycean cysts, heliozoans, charophytes, and ostracods.

### Testate amoeba case study

Testate amoebae have been previously shown to be indicative of environmental changes influencing the hydrobiological community in alpine lakes (Ndayishimiye et al. 2020). Our results provide evidence that this group can also be used as environmental indicators in floodplain lakes.

**Fig. 6:**
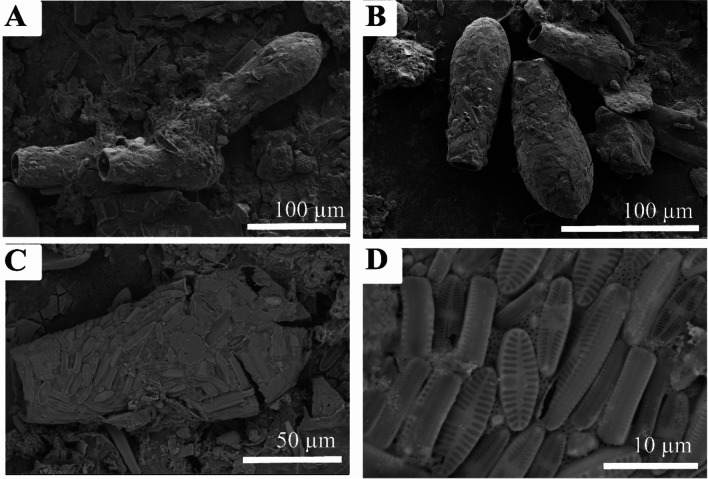
Photomicrographs of *Difflugia*. (**A** and **B**) Multiple samples, illustrating typical shape. (**C**) Example of *Difflugia* composed of diatom frustules, (**D**) close up of (**C**)

**Fig. 7:**
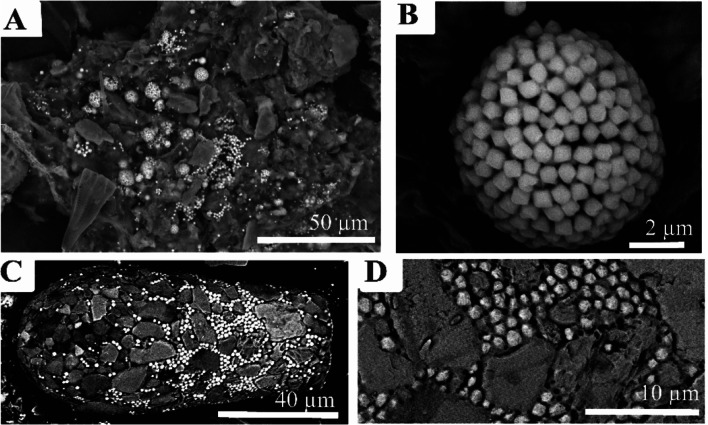
Photomicrographs illustrating the occurrence and appearance of pyrite from Gore Glen. (**A**) Typical view of filter paper showing framboids and individual crystals. (**B**) Close up of individual framboid. (**C**) Example of *Difflugia* utilising individual crystals of pyrite between detrital siliceous grains, (**D**) detail of (**C**)

**Fig. 8:**
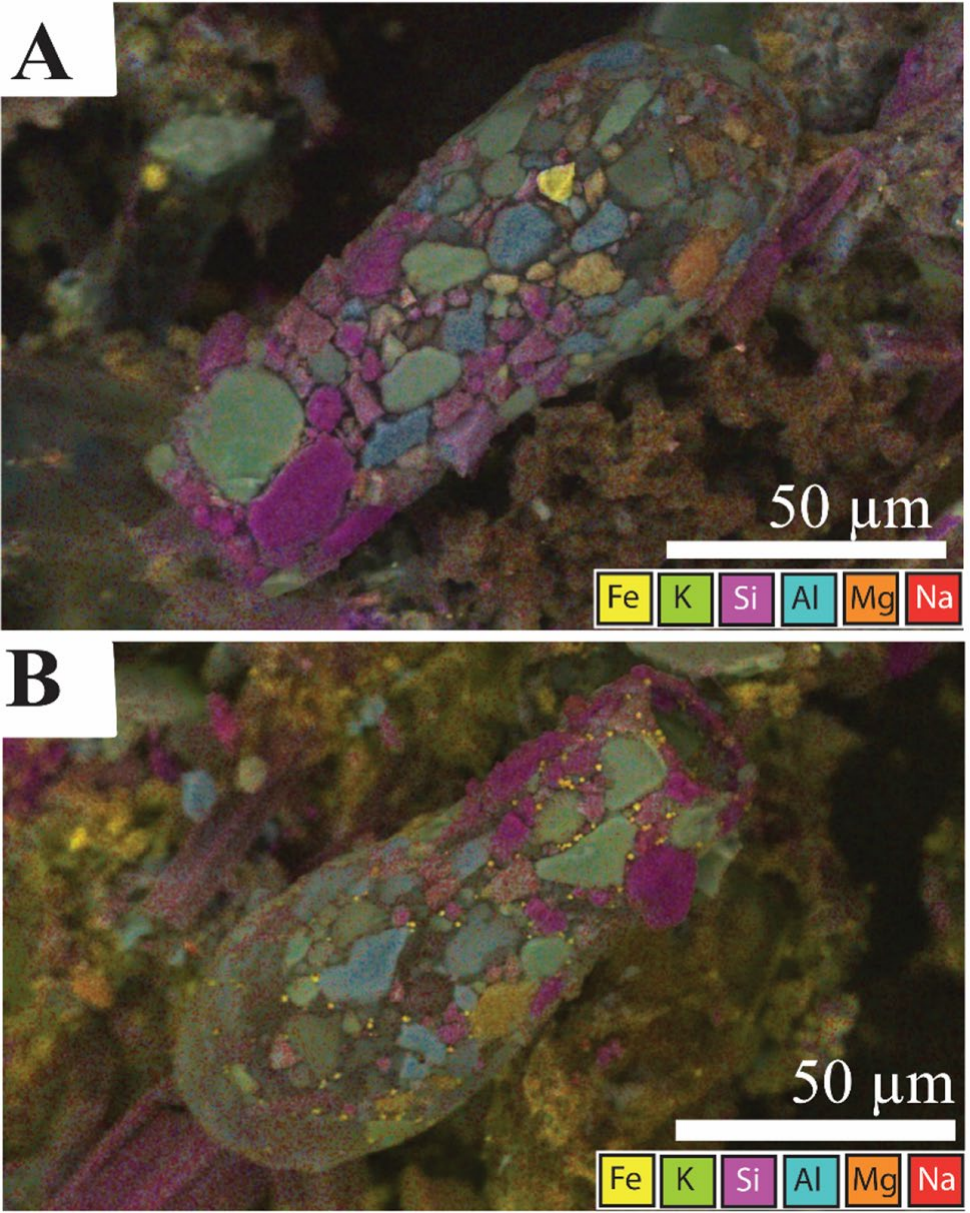
Composite energy-dispersive X-ray maps superimposed on backscattered electron (BSE) SEM micrographs of *Difflugia*, with iron (Fe), potassium (K), silica (Si), aluminium (Al), magnesium (Mg), and sodium (Na). Purple = quartz, blue = feldspar or mica, green = potassium feldspar, orange = magnesium rich silicate, and yellow = pyrite

The testate amoeba at Gore Glen are dominated by the occurrence of *Difflugia* (Fig. 6), whose abundance at the peak of population density in July exceeded that of other testate amoebae by at least an order of magnitude. *Difflugia* almost certainly occurs as more than one species, with various shapes of vase-like forms, with a rounded to pointed fundus, and with a wide range of detrital sediment grain types and arrangements forming the test; incorporating silicate and sulphide grain types and siliceous zooplankton (Figs. 7 and 8).

**Fig. 9:**
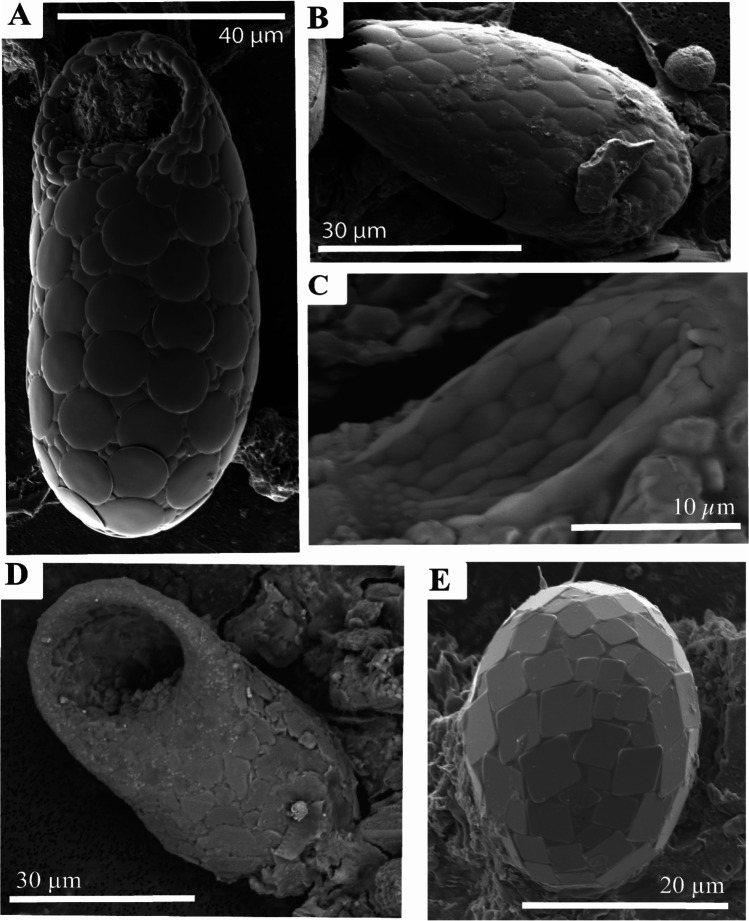
Photomicrographs of other testate amoeba present at Gore Glen. (**A**) *Trinema* sp., (**B**) *Euglypha* sp., (**C**) *Trachelocorythion pulchellum* (forming part of a *Difflugia* test), (**D**) *Centropyxis* sp., (**E**) *Paraquadrula* sp.

In addition to *Difflugia*, there were other testate amoebae observed in the pond samples, albeit their occurrences were rather rare. These included minor occurrences of *Trinema* (4 samples) and *Euglypha* (3 samples), one individual of *Centropyxis*, and one example of *Trachelocorythion pulchellum*, with the latter incorporated into the test of *Difflugia* (Fig. 9). In addition, one example of the calcite plate bearing *Paraquadrula* was also noted (Fig. 9e). Hence, although six genera were recorded during the present study, diversity is generally low, being dominated in terms of volume of individuals by *Difflugia*. This is different to many other local Scottish ecosystems, which are more often dominated by species of siliceous secreting, plate bearing genera such as *Trinema*, *Euglypha*, and *Tracheleuglypha* (pers obs. JB).

**Fig. 10:**
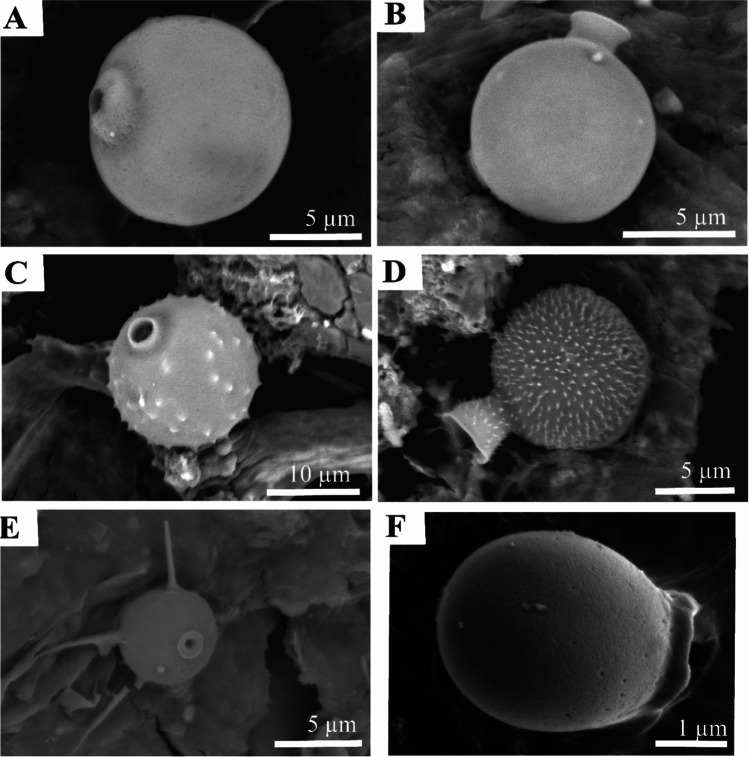
Photomicrographs illustrating the variability of siliceous chrysophycean cysts. (**A** and **B**) Smooth with different apertures. (**C** and **D**) With variable spikey ornamentation and different styles of aperture. (**E**) With long horns. (**F**) Ovate with broad aperture

The occurrence of *Difflugia* within the Gore Glen pond supports previous work indicating the potential use of *Difflugia* as eutrophic indicators and is here shown to proliferate under hypoxic conditions (Fig. 4), being particularly abundant during the Jul–Oct period (observations using SEM and/or light microscopy). The origin of the decomposing organic matter is both autochthonous and allochthonous. Hypoxic conditions may in part have been driven by the decay of plant remains and blooms associated with diatoms (evidenced by decrease in Si levels), cyanobacteria, and the occurrence of golden-brown algae. The latter noted only occasionally by direct observations using light microscopy, but inferred from the occurrence of siliceous chrysophycean cysts (Sandgren 1991) in SEM samples (Fig. 10).

#### Discussion

The results presented in this paper help to provide understanding of the overall patterns of ecosystem functioning of the pond studied. The major factors defining the ecological patterns observed relate to the combination of geomorphological and hydrological settings, with the pond occupying a disused colliery within the floodplain of the Gore Water but directly hydraulically connecting to the river only for events larger than 1 in 5 years. For these events, the pond acts as an online storage feature and attenuates the flow, thus contributing towards the mitigation of downstream flooding. These extreme events are also a potential source of the large amount of sediments, as well as adsorbed chemicals, accumulated in the pond.

Monitoring of chemical and physical variables within the pond provides an insight into the relevant environmental processes and geochemical cycling (Ahilan et al. 2019; Krivtsov et al. 1999, 2001b, 2002, 2003, 2005, 2008a, b, 2009; Tien et al. 2002). The monitoring shows the biological community is principally characterised by the association of the vascular plant *Lemna minor* with cyanobacteria (*Microcystis*) and testate amoebae (dominated by *Difflugia*), both of the latter thriving under eutrophic conditions and nutrient release from sediments due to anoxia. The release of chemicals from sediments is known to be mediated by the drop in pH and oxygen resulting from decomposition of the accumulated organic matter (Krivtsov and Sigee 2005). It should be noted, however, that the pond studied is well-buffered against changes in pH, which is evidenced by relatively high levels of Kh, Gh, and the concentrations of Ca and Mg. Consequently, no dramatic decrease of pH in the surface water was observed (data not shown) although we expect that reductions of pH would have been considerable in the interstitial water (not monitored in this research).

**Fig. 11:**
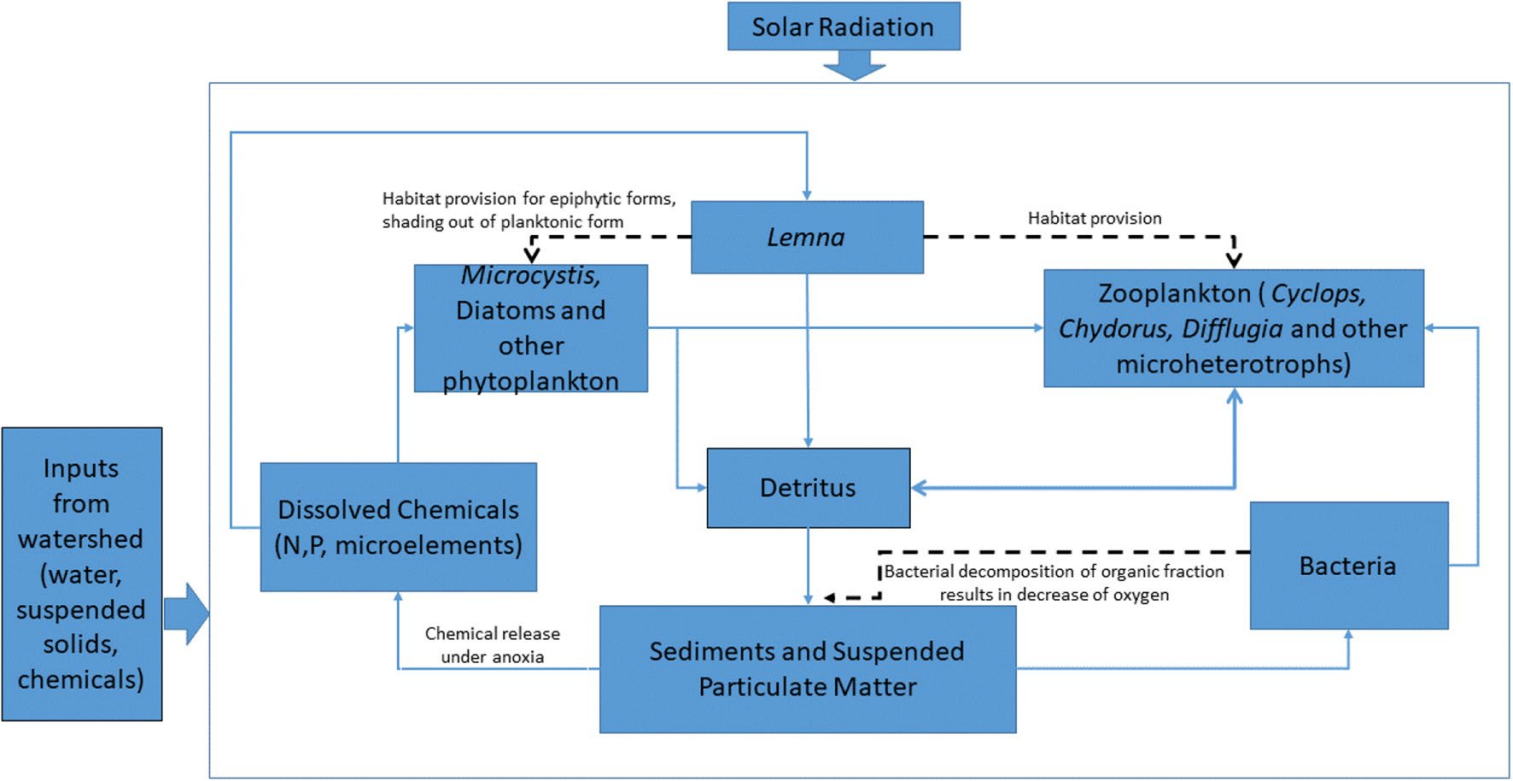
Summary of the key interactions determining the state of the Gore Glen pond ecosystem during summer anoxia. Solid arrows represent flows of energy and matter. Dashed arrows represent other influences

Microscopic observations combined with chemical analysis form objective evidence for describing the interactions amongst biota and biogeochemical cycling. The main interactions that are important for the understanding of the *Lemna/Microcystis/Difflugia* association are summarised in Fig. 11. It should be noted that although there are a number of planktonic species present, the current conditions appear to be particularly favourable for *Microcystis* and *Difflugia*. It is also worth pointing out that this diagram is not intended as an exhaustive account of flows of energy and matter, but aims to depict the key processes crucial for the understanding of the relationships discussed.

The understanding of this biological association and the processes involved is helpful for interpreting the occurrence of other biological organisms as well as issues related to environmental management. For example, although the pond has a thriving population of palmate newts *Lissotriton helveticus*, it may not be feasible to try to introduce *Triturus cristatus* (a newt species important for conservation) due to its more strict ecological requirements (McInerny et al. 2016). Likewise, members of the public would like to see the reduced coverage of duckweed and the presence of such groups as damselflies (whose larvae would require a better water quality). However, due to the ecological interdependencies described here, the successful management actions in that respect are unlikely, and the management should not attempt to clear the vegetation but rather to disseminate the knowledge and promote the enhanced understanding of the relevant ecological and environmental processes. Correct accounting for interdependencies amongst these processes and understanding of their details are indispensable for the optimal environmental management (Krivtsov et al. 2020b, c).

It should also be noted that as return period for the pond’s reconnection to the river is for events greater than 1 in 5 years, most of the time pond and surrounding area will be functioning as traps for pollutants, thus providing protection for ecosystems downstream. However, such conditions may favour the development of cyanobacteria, evidenced in our results by the regular occurrence of *Microcystis*. Thus, dissemination of knowledge by local authorities should also address the presence of *Microcystis* and potential concerns related to its production of toxins.

#### Conclusions

This study has considered the Gore Glen pond located in the forested floodplain of the Gore River. The dynamics of the pond are driven by the thick layer of unconsolidated sediments, which have accumulated a large storage of pollutants. Hydrodynamic modelling shows the pond reconnects with the river only for relatively rare flood events and then acts as a source of sediments and associated pollutants

The state of this eutrophic pond ecosystem is characterised by the association of duckweed *Lemna*, cyanobacterium *Microcystis*, and testate amoebae *Difflugia*. In the summer/early autumn period, practically all of the open water area is covered by duckweed, *Lemna minor* L., which restricts light penetration and oxygen becomes depleted due to decomposition of organic materials. This causes nutrient release from sediments and an increase in water levels of certain elements (e.g., P, Al, Fe, and Mn). These conditions also result in a macroinvertebrates diversity that is impoverished (WHPT index = 16.5). However, both *Difflugia* and *Microcystis* are thriving. Given the potential importance of testate amoebae for paleogeographical reconstructions and cyanobacteria for water quality, this work serves as a useful reference for a wide range of further research and practical applications.

Overall, the measurements and modelling have highlighted the importance and underappreciated multiple benefits provided by ponds located in riverine floodplain woodlands. The study has also revealed complex multivariate interdependencies amongst geomorphology, hydrology, hydrochemistry, and biological variables. The discussion shows that from an environmental viewpoint, the management of the pond is satisfactory but there needs to be improved dissemination of information to the public to promote their enhanced understanding of the relevant ecological and environmental processes. The present study is, therefore, relevant both for the fine-tuning of future management of this important LBS and the country park, as well as more generally for riverine floodplains to ensure that they provide maximum benefits for the public.

### Supplementary Information

ESM 1 (MP4 1.55 mb)

## Data Availability

Data are available for scientific cooperation.

## Appendix 1

Table 2 here

**Table 2:** List of vascular plants recorded at Gore Glen pond

*Acer pseudoplatanus*	
*Agrostis capillaris*	
*Ajuga reptans*	
*Alliaria petiolata*	
*Allium paradoxum*	
*Allium ursinum*	
*Alnus glutinosa*	
*Angelica sylvestris*	
*Anthriscus sylvestris*	
*Athyrium filix-femina*	
*Betula pendula*	
*Brachypodium sylvaticum*	
*Bromopsis ramosa*	
*Cardamine flexuosa*	
*Chamerion angustifolium*	
*Chrysosplenium oppositifolium*	
*Circaea lutetiana*	
*Conopodium majus*	
*Crataegus monogyna*	
*Dactylis glomerata*	
*Deschampsia cespitosa*	
*Dryopteris affine*	
*Dryopteris dilatata*	
*Dryopteris filix-mas*	
*Elymus caninus*	
*Epilobium hirsutum*	
*Epilobium montanum*	
*Epipactis helleborine*	
*Fagus sylvatica*	
*Ficaria verna*	
*Filipendula ulmaria*	
*Fragaria vesca*	
*Fraxinus excelsior*	
*Galium aparine*	
*Galium odoratum*	
*Geranium robertianum*	
*Geum rivale*	
*Geum urbanum*	
*Glechoma hederacea*	
*Glyceria fluitans*	
*Hedera helix*	
*Heracleum sphondylium*	
*Ilex aquifolium*	
*Juncus inflexus*	
*Lapsana communis*	
*Lathraea squamaria*	
*Lemna minor*	
*Luzula sylvatica*	
*Melica nutans*	
*Mercurialis perennis*	
*Myosotis laxa*	
*Myosotis scorpioides*	
*Myosotis sylvatica*	
*Oxalis acetocella*	
*Petasites hybridus*	
*Phalaris arundinacea*	
*Phragmites australis*	
*Poa nemoralis*	
*Poa trivialis*	
*Polystichum setiferum*	
*Primula sp (cf vulgaris)*	
*Prunella vulgaris*	
*Pteridium aquilinum*	
*Ranunculus repens*	
*Rorippa sp*	
*Rosa sp (R canina agg.)*	
*Rubus fruticosus agg*	
*Rumex conglomeratus*	
*Rumex obtusifolius*	
*Rumex sanguinea*	
*Salix caprea*	
*Salix sp (S viminalis)*	
*Sambucus nigra*	
*Silene dioica*	
*Sparganium erectum*	
*Stachys palustris*	
*Stachys sylvatica*	
*Symphytum tuberosum*	
*Taraxacum agg.*	
*Typha latifolia*	
*Typha sp*	
*Ulmus glabra*	
*Urtica dioica*	
*Veronica beccabunga*	
*Veronica montana*	
*Veronica persica*	

## Appendix 2

Table 3 here

**Table 3:** ASPT index values for Gore Glen and other ponds used for comparison

Sampling point	April 2018	June 2018	August 2018	October 2018	January 2019	March 2019	May 2019
Appleton 1	3.9	3.6	4.3	3.6	4.0	3.4	3.9
Appleton 2	3.8	3.8	4.0	3.7	4.0	3.6	3.8
Blackford 1	3.9	3.6	3.6	3.4	3.5	3.3	3.7
Blackford 2	3.7	3.4	3.3	3.3	3.6	3.8	3.6
Eliburn 1	3.8	3.3	3.4	3.3	3.2	2.9	3.5
Eliburn 2	3.6	2.5	2.2	2.6	3.1	3.3	3.5
Gore Glen 1	3.2	3.5	5.5	3.6	3.6	4.2	4.4
Gore Glen 2	3.1	3.6	4.1	1.9	3.9	4.2	4.5
Granton 1	3.3	4.0	3.1	3.0	3.2	3.1	3.8
Granton 2	3.0	3.7	3.2	3.1	3.2	3.6	4.1
Inverleith 1	3.6	4.4	3.3	3.3	3.2	3.3	3.9
Inverleith 2	3.9	3.5	3.4	3.5	2.9	3.8	3.0
Juniper Green 1	4.0	3.6	3.8	3.9	3.7	3.8	3.8
Juniper Green 2	3.4	3.5	4.6	4.1	3.3	3.8	3.8
Oxgangs 1	3.7	3.0	3.4	3.8	3.8	3.4	3.7
Oxgangs 2	3.5	3.7	3.7	3.3	4.2	3.9	3.8
RBGE 1	2.5	3.6	3.5	2.8	2.4	3.1	3.6
RBGE 2	2.8	3.9	3.3	3.2	3.0	3.1	3.6

## Appendix 3

Table 4 here

**Table 4:** WHPT index values for Gore Glen and other ponds used for comparison

Sampling point	April 2018	June 2018	August 2018	October 2018	January 2019	March 2019	May 2019
Appleton 1	43.3	28.6	17.3	17.8	39.5	26.9	58.7
Appleton 2	22.8	30.1	12.1	25.9	43.7	21.4	64.2
Blackford 1	39	51	39.6	37.1	28	42.5	58.9
Blackford 2	40.8	54.3	49.5	49.5	43.7	45.1	57.4
Eliburn 1	37.5	29.3	23.9	22.9	25.5	14.6	31.2
Eliburn 2	21.6	17.8	13	15.7	24.7	23.3	28.3
Gore Glen 1	9.7	10.6	11	7.2	3.6	25.1	30.8
Gore Glen 2	18.4	7.2	12.2	1.9	19.5	42	31.7
Granton 1	19.7	16	18.5	14.8	18.9	12.4	41.5
Granton 2	8.9	29.7	25.4	18.8	25.4	24.9	37.1
Inverleith 1	47.4	21.8	29.9	23.3	12.7	26.4	54.4
Inverleith 2	54.5	24.4	50.7	24.6	14.7	52.6	18
Juniper Green 1	47.6	29	37.9	30.8	33	45.4	53.5
Juniper Green 2	27.1	41.4	9.1	28.8	29.7	57.5	45.4
Oxgangs 1	47.5	15.1	27.4	26.9	23	30.7	44.2
Oxgangs 2	31.1	48.6	36.7	29.6	29.5	35.5	45.3
RBGE 1	4.9	18.1	24.8	8.5	4.8	15.6	25
RBGE 2	8.5	35.2	22.9	12.8	15.1	15.6	29
